# Oral Anticoagulation Choice and Dosage in Very Elderly Patients with Atrial Fibrillation

**DOI:** 10.3390/jcdd12030086

**Published:** 2025-02-26

**Authors:** Martha Zergioti, Melina Kyriakou, Andreas S. Papazoglou, Anastasios Kartas, Dimitrios V. Moysidis, Athanasios Samaras, Efstratios Karagiannidis, Vasileios Kamperidis, Antonios Ziakas, George Giannakoulas

**Affiliations:** 1 Medical School, Aristotle University of Thessaloniki, 54124 Thessaloniki, Greece; marthazergioti@gmail.com (M.Z.); melinakyriakou@yahoo.gr (M.K.); anpapazoglou@yahoo.com (A.S.P.); tkartas@gmail.com (A.K.); vkamperidis@outlook.com (V.K.); aziakas@auth.gr (A.Z.); 2 424 General Hospital of Thessaloniki, 56429 Thessaloniki, Greece; dimoysidis@gmail.com (D.V.M.); stratoskarag@gmail.com (E.K.); 3 Second Department of Cardiology, Hippokration Hospital of Thessaloniki, 54643 Thessaloniki, Greece; ath.samaras.as@gmail.com; 4 First Department of Cardiology, AHEPA University Hospital of Thessaloniki, 54636 Thessaloniki, Greece

**Keywords:** atrial fibrillation, oral anticoagulation, very elderly, octogenarians, nonagenarians

## Abstract

Background: Selecting the optimal oral anticoagulation (OAC) therapy for elderly patients with atrial fibrillation (AF) remains challenging. Our real-world study investigates clinical factors guiding OAC prescription patterns and compares outcomes between full- and reduced-dose direct-acting oral anticoagulants (DOACs) and vitamin K antagonists (VKAs) in this demographic. Methods: This post hoc analysis of the MISOAC-AF trial focused on hospitalized AF patients aged ≥ 75 years prescribed OAC at discharge. Predictors of VKA and reduced DOAC dosing were identified using adjusted odds ratios (aORs). Cox regression models calculated adjusted hazard ratios (aHRs) for primary (all-cause mortality) and secondary outcomes (stroke, bleeding, AF or heart failure hospitalization, cardiovascular death). Results: Among 450 elderly patients, 63.6% received DOACs and 36.4% received VKAs. Higher CHA2DS2-VASc and HAS-BLED scores and antiplatelet use predicted VKA prescription. Hypertension, prior stroke, and bleeding history favored DOAC use. Advanced age and chronic kidney disease correlated with reduced DOAC dosing. Over a 3.7-year follow-up period, there was no significant difference in all-cause mortality between the DOAC and VKA groups (aHR 0.79, 95% CI 0.58–1.06) or between the full-dose and reduced-dose DOAC groups (aHR 0.96, 95% CI 0.60–1.53). Secondary analyses also did not yield statistically significant results in either comparison. Conclusions: Clinical profile parameters in elderly AF patients predict VKA or DOAC use. Clinical outcomes were similar between different OAC therapies.

## 1. Introduction

Atrial fibrillation (AF) represents the most prevalent form of arrhythmia worldwide, with its incidence escalating notably with advancing age [1]. In adults aged 20 or older, AF prevalence is only 3%, whereas in populations aged 75 or older, it is 9–11%, with further escalation when other cardiac comorbidities exist [2]. Projections suggest that over the forthcoming decade, AF is poised to affect between 14 and 17 million individuals within the European Union [3]. This relative increase is primarily attributed to demographic shifts characterized by an aging population, alongside enhanced detection capabilities and the exacerbating influence of predisposing health conditions such as diabetes mellitus (DM) and hypertension [4].

Over the last decades, direct-acting oral anticoagulants (DOACs) have revolutionized the therapeutic landscape for AF [5]. Since 2016, they have emerged as the preferred first-line oral anticoagulation (OAC) strategy owing to their demonstrated net clinical superiority over vitamin K antagonists (VKAs) [6]. Given the constant aging of the global population and the increasing prevalence of AF in elderly and very elderly populations, it becomes imperative to discern the optimal OAC strategy for these subgroups [7]. Currently, a substantial proportion of elderly AF patients receive suboptimal anticoagulation therapy, primarily due to concerns regarding bleeding risk (attributable to factors such as low body weight, impaired renal function, and frailty), as well as the presence of cardiac or extra-cardiac comorbidities, and non-adherence to therapy [8,9,10].

Consequently, an expanding body of literature endeavors to evaluate OAC and dosage-related outcomes in very elderly AF patients, who are often excluded from clinical trials [10,11,12,13,14,15]. The primary objective of our real-world cohort study, conducted on AF patients enrolled between December 2015 and June 2018, was to provide a succinct overview of OAC-related decision-making in AF patients aged 75 years and older, as observed at discharge by cardiologists in a tertiary hospital, reflecting the clinical practices of that era. Furthermore, our study seeks to contribute to the existing evidence base by addressing the pivotal question of whether full- or reduced-dose DOACs or VKAs offer superior clinical outcomes in the very elderly AF population.

## 2. Materials and Methods

### 2.1. Study Design and Study Population

This study constitutes a retrospective post hoc analysis of the MISOAC-AF randomized trial (Motivational Interviewing to Support Oral Anticoagulation adherence in patients with non-valvular Atrial Fibrillation, ClinicalTrials.gov identifier: NCT02941978) [16]. In brief, the MISOAC-AF trial randomized a diverse cohort of 1140 hospitalized AF patients into two groups, exploring the impact of patient–physician interviews and scripted guidance on adherence to OACs. The trial outcomes have been previously published [17]. The study adhered to the World Medical Association Declaration of Helsinki and received approval from the Bioethics Committee of the Aristotle University of Thessaloniki (protocol code: 173, date of approval: 30 November 2015). Each participant provided written informed consent before participating in the study.

In this particular investigation, we included only the subset of AF patients aged 75 and above, defined as a very elderly group of patients. Individuals with incomplete data regarding their discharge OAC regimen and those not receiving any OACs were excluded from the analysis. The included patients were categorized based on the OAC treatment at discharge, namely VKA (acenocoumarol) and DOACs (dabigatran, rivaroxaban, or apixaban). A full DOAC dose was deemed to be one comprising 150 mg dabigatran, 20 mg rivaroxaban, and 5 mg apixaban, while a reduced DOAC dose comprised 110 mg dabigatran, 15 mg rivaroxaban, and 2.5 mg apixaban.

### 2.2. Study Endpoints

The primary endpoint of the study was the occurrence of all-cause death during follow-up. The secondary endpoints included any stroke, major bleeding, and the composite endpoint of AF- or heart failure (HF)-related hospitalization or cardiovascular death.

### 2.3. Definition of Covariates

AF was identified as either previously documented in the patient’s medical history or as new-onset AF observed during hospitalization. The latter was characterized by an irregular heart rhythm persisting for more than 30 s, lacking detectable P waves, as captured by a 12-lead electrocardiogram or a 24 h Holter monitor. Cardiovascular death was defined as a death related to cardiac causes or stroke. Stroke was defined as the emergence of new, focal, or global neurological dysfunction resulting from vascular injury in the brain, spinal cord, or retinal region due to infarction. Stroke criteria included a symptom duration of at least 24 h, or otherwise the presence of a clearly corresponding lesion on CT or MRI scans. Major bleeding was characterized by fatal bleeding, symptomatic hemorrhages in critical areas or organs, a reduction in hemoglobin levels by at least 2 g/dL, or the requirement of a transfusion of at least 2 units of whole blood or red cells.

### 2.4. Statistical Analysis

Continuous variables are presented as means with standard deviations (SDs), while categorical variables are presented as numbers with percentages. Between-group comparisons of the categorical variables were performed using the chi-square test, while the t-test was utilized for the comparison of continuous variables.

Univariate and multivariate binary logistic regression analyses were performed to identify potential predictors of VKA prescription as compared to DOACs (used as the reference category). The variables forced into the multivariate model were selected based on their clinical relevance and the univariate logistic regression outcomes (i.e., we included the variables with univariate *p*-value < 0.20).

Survival analyses, accompanied by time-to-event plots (Kaplan–Meier curves), were conducted for each study endpoint to compare the prognostic course of patients receiving VKAs and DOACs (full vs. reduced dose). A multivariate Cox regression hazard model was developed by incorporating variables of clinical interest or those univariately associated with each endpoint (*p* < 0.20) into the multivariate analysis. These variables included age, gender, body mass index (BMI), history of smoking, HF, coronary artery disease (CAD), chronic kidney disease (CKD), DM, hypertension, stroke, and the CHADS-VASc score. Sensitivity multivariate regression analyses were also performed to include as covariates the eGFR levels at discharge and the rates of anticoagulation adherence during follow-up [18]. A two-tailed significance threshold was set at a = 0.05. The statistical analysis was performed using SPSS version 25 (SPSS Inc., Chicago, IL, USA) software.

## 3. Results

### 3.1. Baseline Characteristics

A total of 450 (39.5% of the MISOAC-AF trial population) patients with AF (47.3% males) were treated with OACs and were, thereby, included in this analysis, with a mean age of 81.9 ± 4.3 years. Of them, 286 (63.6%) received a DOAC (59 dabigatran, 103 rivaroxaban, 124 apixaban), and 164 (36.4%) a VKA (acenocoumarol). Patients on DOACs had lower HAS-BLED and CHA2DS2-VASc scores than patients on VKA (*p* < 0.01). Higher glomerular filtration rates (GFR) were observed in the DOAC group (*p* = 0.02). Higher rates of prior HF and CAD were documented in the VKA group (*p* < 0.05).

Regarding the dose of DOACs, 190 patients (66.4% of those under DOACs) received a reduced dose. Between the two subgroups (full vs. reduced DOAC dose), there were significant differences in age, male %, BMI, creatinine, GFR, and the prevalence of peripheral artery disease, AMI, and CKD. Baseline patient characteristics according to OAC status (DOACs vs. VKA) and DOAC dose (full vs. reduced dose) are depicted in detail in Table 1 and Table 2, respectively. The subcategories of the type and dosage of OACs are depicted in Supplementary Table S1.

### 3.2. Prediction of OAC Discharge Prescription

The binary logistic regression analysis set for the association of baseline covariates with DOAC or VKA prescription yielded that increasing CHA2DS2-VASc score (aOR 0.69, 95% CI 0.50–0.95, *p* 0.02) and HAS-BLED score (aOR 0.33, 95% CI 0.23–0.48, *p* < 0.01) were both correlated with a higher probability of VKA prescription. Additionally, the prescription of concomitant antiplatelet medication was negatively linked with DOAC prescription (aOR 0.74, 95% CI 0.54–1.00, *p* 0.05). On the other hand, comorbid hypertension (aOR 4.80, 95% CI 2.30–10.00, *p* < 0.01), prior stroke (aOR 4.90, CI 1.80–13.44, *p* < 0.01), and major bleeding (aOR 2.72, 95% CI 1.26–5.88, *p* 0.01) were independently linked with increased likelihood of DOAC prescription. A detailed overview of these results can be found in Table 3. 

### 3.3. Association of OAC Type (DOACs vs. VKA) with Clinical Outcomes

The primary endpoint of all-cause death occurred in 203 (45.1%) patients over a median 3.7-year follow-up period. Among the deceased patients, 113 (39.5%) received a DOAC, and 90 (54.9%) a VKA at hospital discharge (Table 4). According to the univariable survival analysis, a lower risk of all-cause death was observed in patients on DOACs (HR 0.69, 95% CI 0.51–0.88; log-rank test: *p* < 0.01), compared to patients on VKAs. However, after adjustment for potential confounders, the risk for all-cause death did not differ significantly between the two groups (aHR 0.79, 95% CI 0.58–1.06, *p* 0.12).

**Figure 1:**
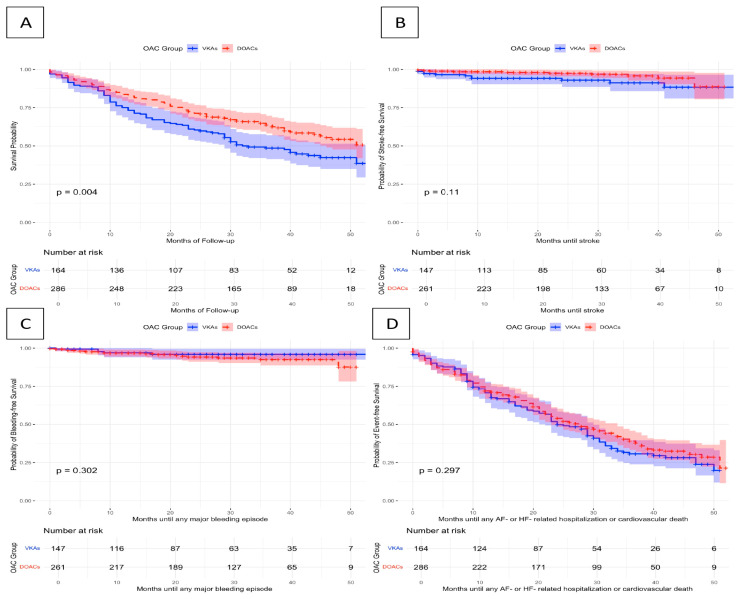
Kaplan–Meier curves plotted to illustrate the association of VKAs or DOACs with the occurrence of any: (**A**) all-cause mortality, (**B**) stroke, (**C**) major bleeding episode, (**D**) AF- or HF-related hospitalization or cardiovascular death.

Moreover, incidence of bleeding and stroke were investigated as secondary endpoints along with the composite endpoint of cardiovascular mortality and any HF- or AF-related hospitalization. Both adjusted and unadjusted risks for the secondary endpoints did not differ significantly between patients under VKA and those under DOACs. A detailed overview of these endpoints is displayed in Table 4 and Figure 1. The sensitivity analysis including OAC follow-up adherence as a covariate did not alter the prognostic effects of VKA vs. DOACs (Supplementary Table S2).

### 3.4. Prediction of the Prescription of Full vs. Reduced DOAC Dose

In total, 190 (66.4%) study participants were administered a reduced DOAC dose and 96 (33.6%) a full DOAC dose. The prescription of a reduced DOAC dose was positively correlated with increasing age (aOR 1.09, 95% CI 1.01–1.17, *p* 0.03) and history of CKD (aOR 3.53, 95% CI 1.92–6.49, *p* < 0.01). Patients with prior AMI showed a tendency to be administered a reduced DOAC dose (aOR 3.01, 95% CI 0.92–9.88, *p* 0.07); however, this analysis did not reach the statistical significance threshold. A detailed overview of these results can be found in Table 5. The sensitivity analysis including eGFR levels at discharge as a covariate instead of the CKD history did not alter the significance of the remaining predictors and yielded that increasing eGFR leads to a decreased likelihood of prescription of reduced-dose DOACs (aOR 0.96, 95% CI 0.95–0.98). 

### 3.5. Association of DOAC Dose (Full vs. Reduced) with Clinical Outcomes

**Figure 2:**
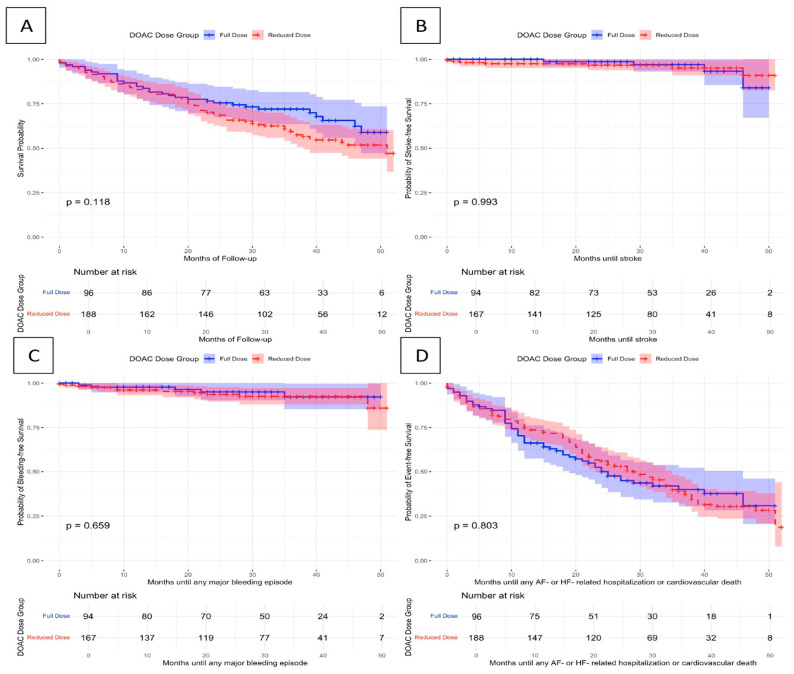
Kaplan–Meier curves plotted to illustrate the association of full or reduced DOAC dose with the occurrence of any: (**A**) all-cause mortality, (**B**) stroke, (**C**) major bleeding episode, (**D**) AF- or HF-related hospitalization or cardiovascular death.

Among the deceased patients, 81 received a reduced DOAC dose (43.1%) and 32 a full DOAC dose (33.7%) at hospital discharge (Table 6). According to the univariable survival analysis, there was no difference in the risk of all-cause mortality in patients receiving reduced vs. full DOAC doses (HR 1.32, 95% CI 0.88–1.99, log-rank test: *p* 0.12; Figure 2). This did not alter after adjustment for confounding factors (aHR 0.96, 95% CI 0.60–1.53, *p* 0.85).

Moreover, incidence of bleeding, stroke, and the composite endpoint of any HF- or AF-related hospitalization or cardiovascular death were investigated as secondary endpoints. Both adjusted and unadjusted analyses for those endpoints did not show significantly different risk between patients under reduced and full DOAC doses. A detailed overview of these endpoints is displayed in Table 6 and Figure 2. The sensitivity analysis including OAC follow-up adherence as a covariate did not alter the prognostic effects of a full vs. reduced DOAC dose (Supplementary Table S2).

## 4. Discussion

In this post hoc observational analysis derived from the MISOAC-AF randomized trial, our investigation centered on evaluating prescription patterns and clinical outcomes associated with various OAC strategies among elderly individuals aged 75 years and older with concomitant AF. The mean age of the participants enrolled in our study was 81.9 years, with a corresponding mean CHA2DS2-VASc score of 5.4, indicative of a very high-risk real-world AF population. Notably, higher CHA2DS2-VASc and HAS-BLED scores, alongside concurrent antiplatelet therapy, were observed to correlate with an augmented likelihood of VKA prescription upon discharge. Conversely, the presence of comorbidities such as hypertension, prior stroke, and major bleeding events were associated with a higher likelihood of DOAC prescription. Furthermore, advancing age and comorbid CKD emerged as independent predictors of a higher prevalence of reduced-dose DOAC prescriptions compared to full-dose regimens. However, our comparative analysis revealed similar rates of adverse cardiovascular events during follow-up when contrasting DOACs with VKAs and full-dose DOACs with reduced-dose DOACs.

Aging is known to be associated with multiple comorbidities, polypharmacy, and altered drug pharmacokinetics [19]. Furthermore, it is considered an independent risk factor for both bleeding and thromboembolic events [20]. This dual increase in bleeding and thrombotic risk in the elderly may lead clinicians to experience uncertainty regarding the optimal anticoagulation regimen. Overall, compared with warfarin, DOACs exhibit a favorable risk–benefit profile, and their efficacy and safety in elderly patients are consistent with those of the overall population [21,22,23,24]. Because DOACs have a wide therapeutic window, predictable anticoagulant effects, and few interactions with other drugs, they may be preferable to VKAs, and their prescription is recommended in the latest guidelines (Class IA, 2023 ACC/AHA/ACCP/HRS Guideline for the Diagnosis and Management of AF) [25].

The primary survival analysis yielded a non-significant trend of higher mortality in patients on VKAs compared to patients on DOACs, which could be attributed to the increasing frailty and complicated status of patients on VKAs. Nevertheless, no statistical superiority of either VKAs or DOACs was proved after adjustment, and the clinical superiority of DOACs over VKAs was not confirmed in this multimorbid AF population. A comprehensive review of the latest literature reveals the safety and efficacy of DOACs as an anticoagulation regimen in the elderly. For example, the prospective multicenter START2-REGISTER study, which included AF patients aged ≥85 years, indicated a significant survival benefit in those treated with DOACs with similar bleeding risk but a higher risk for cerebral thrombotic events compared to VKAs [26]. Moreover, a Norwegian nationwide cohort study of elderly AF patients initiating thromboprophylaxis showed that both full and reduced DOAC doses were associated with similar risks of thromboembolism to those of warfarin and lower or similar risks of bleeding [27]. Subgroup analyses of the RE-LY trial [28], the ROCKET-AF trial [22], and the ARISTOTLE trial [29] also demonstrated the clinical benefit and safety of DOAC use in patients aged ≥75 years. However, a recent randomized trial (the FRAIL-AF) concluded that switching from VKAs to DOACs in frail older patients with AF was associated with more bleeding complications, without a reduction in thromboembolic complications, compared with continuing VKA treatment [30]. Nevertheless, a recent study conducted in Sichuan, China reached a different conclusion regarding elderly, frail patients with AF and highlighted the importance of DOAC therapy to reduce risk of thrombotic events; at the same time, there was no increase in fatal bleeding [31].

Choosing the appropriate dosing of DOACs is a complex question for most physicians, especially when treating elderly populations, where they must consider competing risks of stroke and bleeding, anticipated changes in renal function, interacting medications, and patient frailty to establish an appropriate starting dose. These difficulties are compounded by the exclusion or underrepresentation of the most complex cases, such as patients with severe CKD or prior intracranial bleeding, in pivotal DOAC trials. The possibility of underdosing or overdosing should also be taken into account. A recent study from the Global Anticoagulant Registry in the FIELD-AF (GARFIELD-AF) investigated the degree of recommended and non-recommended dosing of DOACs among 10,426 AF patients and found that 23.2% were underdosed and 3.8% were overdosed. Interestingly, the prescription of non-recommended doses was associated with a 25% higher risk of all-cause mortality. Similarly, the PAVE-AF study (Tzeis et al.) also revealed reluctance in DOAC prescription or prescription with inappropriate dosing in elderly population; the EUROSAF study also highlighted the underuse of optimal DOAC dosing despite the preference of DOACs over VKAs, concluding that significant treatment gaps may exist, driven by multimorbidity, polypharmacy, and concerns about bleeding risks [32,33,34,35].

### Limitations

The primary limitation of this study is its retrospective design. Τhe presence of unmeasured confounders that may have influenced the choice of OAC treatment in this subgroup is highly likely. While we adjusted for baseline characteristics with clinical and statistical significance, the lack of information on follow-up changes in anticoagulation treatment or INR/eGFR levels during follow-up could lead to misleading outcomes. No propensity-score matching approach was implemented in this study to reduce potential treatment selection bias. Moreover, there were no frailty indices documented to assess patient stratification and explore the role of frailty in anticoagulation decision. Furthermore, the limited sample size of this study resulted in analyses with insufficient power to assess hard clinical endpoints and prevented subgroup analyses based on DOAC type. Consequently, our findings should be considered exploratory.

The data, as they were extracted from the MISOAC-AF trial, reflect an older time period in terms of recommended anticoagulation patterns; the first enrollment and randomization took place in December 2015, and subsequent recruitment in MISOAC-AF was completed in June 2018. The timing of the study may possibly explain the non-significant trend of higher mortality observed in patients on VKAs in the univariate survival analysis. During that period, doctors tended to prescribe VKAs to patients with more complex AF, a practice that may no longer be common today. Finally, our sample size may not have been sufficient to replicate the previously reported results from other trials. It is important to highlight the small number of patients divided into either of the two subgroups (190 vs. 96 patients under the full and reduced DOAC dose, respectively) in our study. However, the lack of large randomized controlled trials comparing DOACs head-to-head at different dosages, and the difficulty of conducting future large-scale clinical trials investigating outcomes associated with different doses, should also be taken into account; such trials are hardly feasible, as deliberately underdosing or overdosing patients in a clinical trial is ethically problematic.

## 5. Conclusions

This observational post hoc analysis of a randomized clinical trial explored prescription patterns and clinical outcomes of OAC strategies in very elderly patients aged over 75 years with comorbid AF. The study population exhibited a high-risk profile which is indicative of a real-world AF cohort. In this population, the survival benefit of DOACs over VKAs was not confirmed. The intricacies of dosing remain a clinical conundrum, particularly in elderly populations with complex medical histories. Individualized dosing decisions should consider factors such as renal function, concurrent medications, past medical history, and frailty. Larger-scale observational studies and randomized controlled trials are needed to compare the effects of DOACs at different dosages in this clinical context.

## Figures and Tables

**Table 1:** Baseline characteristics according to oral anticoagulation scheme prescribed.

	All Patients Under OACs (n = 450, 100%)	Patients Under VKA (n = 164, 36.4%)	Patients Under DOAC (n = 286, 63.6%)	*p*-Value
***Demographics***				
Male gender, n (%)	213 (47.3)	77 (47)	136 (47.6)	0.90
Age, mean (SD)	81.9 (4.3)	81 (4.2)	81.2 (4.3)	0.85
***Type of AF***				
Temporal type of AF, n (%)	445	163	282	
First diagnosed AF, n (%)	40 (9)	10 (2.2)	30 (6.7)	0.29
Paroxysmal AF or atrial flutter, n (%)	137 (30.8)	42 (9.4)	95 (21.3)	0.29
Persistent or permanent AF, n (%)	268 (60.2)	111 (24.9)	157 (35.3)	0.29
***Cardiovascular risk factors and comorbidities***				
BMI (kg/m^2^), mean (SD)	28 (4.8)	27.6 (4.9)	28 (4.8)	0.84
History of smoking (prior or current), n (%)	234 (53.3)	84 (52.2)	150 (54)	0.72
Serum creatinine (mg/dL), mean (SD)	1.33 (0.88)	1.37 (0.86)	1.16 (0.41)	<0.01
CHA2DS2-VASc score, mean (SD)	5.4 (1.4)	5.8 (1.6)	5.3 (1.4)	<0.01
HAS-BLED score, mean (SD)	12 (0.9)	2.43 (1.0)	1.7 (0.8)	<0.01
HAS-BLED ≥ 3, n (%)	118 (26.2)	77 (47)	41 (14.3)	<0.01
Estimated GFR with Cockcroft–Gault formula, mean (SD)	27.5 (29.69)	24 (27)	28.6 (30.8)	0.02
Estimated GFR with CKD-EPI formula, mean (SD)	53.4 (19.9)	51.9 (19.5)	56.3 (18.3)	0.43
Hemoglobin (g/dL), mean (SD)	12.30 (2.32)	12.3 (2.3)	12.4 (2.5)	0.29
Coronary artery disease, n (%)	270 (60.9)	117 (71.8)	153 (54.6)	<0.01
Heart failure, n (%)	270 (60.9)	117 (71.8)	153 (54.6)	<0.01
Prior myocardial infarction, n (%)	270 (60.9)	117 (71.8)	153 (54.6)	<0.01
Pacemaker/ ICD, n (%)	48 (2.5)	25 (5.7)	23 (5.2)	0.07
Hypertension, n (%)	389 (87.4)	131 (80.9)	258 (91.2)	<0.01
Dyslipidemia, n (%)	218 (49.2)	86 (52.8)	132 (47.1)	0.25
Diabetes mellitus, n (%)	165 (37.1)	68 (41.7)	97 (34.4)	0.12
Peripheral artery disease, n (%)	217 (49.3)	95 (59)	122 (43.7)	<0.01
Prior ischemic stroke (%)	57 (12.8)	25 (15.5)	32 (11.2)	0.19
Prior systemic thromboembolic disease, n (%)	38 (8.5)	19 (11.8)	19 (6.6)	0.06
Prior intracranial bleeding, n (%)	3 (0.7)	2 (1.2)	1 (0.4)	0.27
Prior upper gastrointestinal bleeding, n (%)	35 (7.9)	14 (8.7)	21 (7.4)	0.62
Prior lower gastrointestinal bleeding, n (%)	36 (8.1)	13 (2.9)	23 (8.1)	0.99
Prior bleeding episodes, n (%)	147 (33)	57 (35.4)	90 (31.7)	0.42
Chronic kidney disease, n (%)	269 (60.6)	107 (66)	162 (57.4)	0.07
Hepatic disease, n (%)	9 (2.1)	3 (1.9)	6 (2.2)	0.83
Thyroid disease, n (%)	96 (21.8)	32 (19.9)	64 (22.9)	0.45
***Medication***				
Use of rate control medication at discharge (b-blocker, digoxin, or both), n (%)	362 (80.4)	128 (78)	234 (81.8)	0.33
Use of antiplatelets at discharge (aspirin, clopidogrel, or both), n (%)	75 (17.1)	49 (29.9)	26 (9.5)	<0.01
Use of rhythm control medication at discharge (propafenone, amiodarone, sotalol), n (%)	79 (17.6)	27 (16.5)	52 (18.2)	0.65

**Table 2:** Baseline patient characteristics according to DOAC dose (full vs. reduced).

	All Patients Under DOACs (N = 286, 100%)	Reduced Dose of DOACs (N = 190, 66.4%)	Full Dose of DOACs (N = 96, 33.6%)	*p*-Value
***Demographics***				
Male gender, n (%)	134 (46.9)	97 (51.1)	37 (38.5)	0.04
Age (years), mean (SD)	81.2 (4.3)	81.9 (4.4)	79.9 (3.7)	<0.01
***Type of AF***				
First diagnosed AF, n (%)	30 (10.8)	15 (16)	15 (8.1)	0.12
Paroxysmal AF or atrial flutter, n (%)	94 (33.7)	66 (35.7)	28 (29.8)	0.12
Persistent or permanent AF, n (%)	155 (55.6)	104 (56.2)	51 (54.3)	0.12
***Cardiovascular risk factors and comorbidities***				
BMI (kg/m^2^), mean (SD)	28.0 (4.8)	27.6 (4.7)	28.9 (4.8)	0.03
History of smoking (prior or current), n (%)	148 (53.8)	106 (58.2)	42 (45.2)	0.04
Serum creatinine (mg/dL), mean (SD)	1.16 (0.4)	1.2 (0.4)	1 (0.3)	<0.01
CHA2DS2-VASc score, mean (SD)	5.3 (1.4)	5.3 (1.3)	5.2 (1.4)	0.47
HAS-BLED score, mean (SD)	1.7 (0.8)	1.7 (0.8)	1.7 (0.8)	0.94
HAS-BLED ≥ 3, n (%)	41 (14.5)	29 (15.4)	12 (12.6)	0.53
Estimated GFR with Cockcroft–Gault formula, mean (SD)	28.6 (30.8)	29.2 (29.2)	27.1 (34.0)	0.59
Estimated GFR with CKD-EPI formula, mean (SD)	56.3 (18.3)	52 (17.7)	64.5 (16.5)	<0.01
Hemoglobin (g/dL), mean (SD)	12.4 (2.5)	12.4 (2.7)	12.4 (2.0)	0.91
Coronary artery disease, n (%)	91 (32.4)	72 (38.7)	19 (20.0)	<0.01
Heart failure, n (%)	164 (58)	116 (61.7)	48 (50.5)	0.07
Prior myocardial infarction, n (%)	48 (17.3)	41 (22.3)	7 (7.5)	<0.01
Pacemaker/ ICD, n (%)	23 (8.3)	17 (9.2)	6 (6.5)	0.28
Hypertension, n (%)	256 (91.4)	169 (90.9)	87 (92.6)	0.63
Dyslipidemia, n (%)	130 (46.9)	84 (45.7)	46 (49.5)	0.55
Diabetes mellitus, n (%)	97 (34.8)	62 (33.3)	35 (37.6)	0.48
Peripheral artery disease, n (%)	120 (43.5)	87 (47.5)	33 (35.5)	0.06
Prior ischemic or hemorrhagic stroke or transient ischemic attack, n (%)	47 (16.7)	34 (18.1)	13 (13.8)	0.63
Systemic thromboembolic disease, n (%)	19 (6.7)	14 (7.4)	5 (5.3)	0.49
Prior intracranial bleeding, n (%)	1 (0.4)	1 (0.5)	0 (0.0)	0.47
Prior upper gastrointestinal bleeding, n (%)	21 (7.5)	13 (7.0)	8 (8.4)	0.67
Prior lower gastrointestinal bleeding, n (%)	23 (8.2)	15 (8.1)	8 (8.4)	0.92
Prior bleeding episodes, n (%)	90 (32.0)	56 (30.1)	34 (35.8)	0.33
Chronic kidney disease, n (%)	161 (57.7)	126 (68.1)	35 (37.2)	<0.01
Hepatic disease	6 (2.2)	4 (2.2)	2 (2.2)	0.98
***Medication***				
Use of rate control medication at discharge (b-blocker, digoxin, or both), n (%)	231 (81.6)	152 (80.9)	79 (83.2)	0.90
Use of antiplatelets at discharge (aspirin, clopidogrel, or both), n (%)	25 (9.1)	21 (11.4)	4 (4.4)	0.10
Use of rhythm control medication at discharge (propafenone, amiodarone, sotalol), n (%)	52 (18.3)	33 (20)	19 (6.7)	0.35

**Table 3:** Logistic regression outcomes for the prediction of VKA prescription at hospital discharge.

	Univariate *p*-Value	Multivariate *p*-Value	Adjusted OR (95% CIs)
Female gender	0.90	0.94	1.02 (0.55–1.90)
Age	0.63	0.71	0.99 (0.94–1.05)
Smoking (ever)	0.72	0.81	0.94 (0.57–1.55)
Body mass index	0.69	0.62	0.99 (0.94–1.04)
CHA2DS2-VASc score	<0.01	0.02	**0.69 (0.50–0.95)**
HAS-BLED score	<0.01	<0.01	**0.33 (0.23–0.48)**
Coronary artery disease	<0.01	0.80	0.93 (0.52–1.66)
Hypertension	<0.01	<0.01	**4.80 (2.30–10.00)**
Diabetes mellitus	0.12	0.24	1.43 (0.79–2.58)
Chronic kidney disease	0.08	0.09	0.64 (0.39–1.07)
**Prior stroke or TIA**	0.05	<0.01	**4.90 (1.8–13.44)**
Prior major bleeding	0.94	0.01	**2.72 (1.26–5.88)**
Antiplatelet use	<0.01	0.05	**0.74 (0.54–1.00)**

**Table 4:** Survival analysis outcomes based on the type of OAC received (VKA vs. DOACs).

Outcome	Rates of Occurrence in VKA	Rates of Occurrence in DOACs	Log-Rank Test *p*-Value	Univariate HR Cis (95%)	Multivariate HR * Cis (95%)
All-cause mortality	90 (54.9)	113 (39.5)	0.004	0.69 (0.50–0.88)	0.79 (0.58–1.06)
CV mortality	69 (42.1)	80 (28.0)	0.003	0.62 (0.45–0.85)	0.76 (0.53–1.07)
Bleeding	5 (3.4)	16 (6.1)	0.302	1.69 (0.62–4.60)	1.50 (0.52–4.27
Stroke	11 (7.5)	11 (4.2)	0.110	0.51 (0.22–1.17)	0.43 (0.17–1.08)
AF- or HF-related hospitalization or CV death	116 (70.7)	163 (57.0)	0.297	0.95 (0.64–1.43)	0.84 (0.63–1.11)

* Cox regression analysis adjusted for age, gender, smoking, BMI, Hx of diabetes mellitus, Hx of HF, Hx of CAD, Hx of hypertension, Hx of CKD, Hx of stroke, and CHADS-VASc score.

**Table 5:** Logistic regression outcomes for the prediction of full vs. reduced DOAC dose prescription.

Covariate	Univariate *p*-Value	Multivariate *p*-Value	Adjusted OR (95% CIs)
Diabetes mellitus	0.477	0.449	0.74 (0.35–1.60)
Age	<0.001	0.033	**1.09 (1.01–1.17)**
Female gender	0.044	0.450	0.73 (0.32–1.65)
Body mass index	0.035	0.969	1.00 (0.94–1.07)
Smoking (ever)	0.040	0.098	1.68 (0.91–3.12)
Heart failure	0.073	0.639	1.20 (0.56–2.56)
Coronary artery disease	0.002	0.730	1.18 (0.46–3.05)
Prior acute myocardial infarction	0.003	0.069	3.01 (0.92–9.88)
Chronic kidney disease	<0.001	<0.001	**3.53 (1.92–6.49)**
History of any stroke or TIA	0.366	0.924	1.07 (0.26–4.45)
History of bleeding	0.335	0.180	0.64 (0.33–1.23)
HAS_BLED > 3	0.528	0.854	1.09 (0.42–2.82)
CHA2DS2_VASC	0.334	0.887	1.04 (0.64–1.67)

**Table 6:** Survival analysis outcomes based on the differentiation of full vs. reduced DOAC dose.

Outcome	Rates of Occurrence in DOAC Reduced Dose	Rates of Occurrence in DOAC Full Dose	Log-Rank Test *p*-Value	Univariate HR (95% CIs)	Multivariate aHR * (95% CIs)
*All-cause mortality*	81 (43.1)	32 (33.7)	0.118	1.32 (0.88–1.99)	0.96 (0.60–1.53)
*CV mortality*	59 (31.4)	21 (22.1)	0.137	1.46 (0.89–2.40)	0.84 (0.47–1.50)
*Bleeding*	11 (6.6)	5 (5.5)	0.659	1.24 (0.43–3.56)	1.32 (0.42–4.16)
*Stroke*	7 (4.2)	4 (4.4)	0.993	0.97 (0.28–3.31)	1.24 (0.27–5.70)
*AF- or HF-related hospitalization or CV death*	93 (49.5)	39 (41.1)	0.803	1.09 (0.75–1.59)	0.79 (0.50–1.22)

* Cox regression analysis adjusted for age, gender, smoking, BMI, Hx of diabetes mellitus, Hx of HF, Hx of CAD, Hx of hypertension, Hx of CKD, Hx of stroke, and CHADS-VASc score.

## Data Availability

Data are available from George Giannakoulas (e-mail: ggiannakoulas@auth.gr) upon reasonable request and with the permission of AHEPA University Hospital.

## Supplementary Materials

The following supporting information can be downloaded at: https://www.mdpi.com/article/10.3390/jcdd12030086/s1, Table S1: Type and dose of OAC prescribed at hospital discharge. Table S2: Sensitivity analysis outcomes after adjustment for patients’ anticoagulation adherence during follow-up.

## List of Abbreviations

OAC: oral anticoagulation, AF: atrial fibrillation, DOACs: direct oral anticoagulants, VKAs: vitamin K antagonists, GFR: glomerular filtration rate, BMI: body mass index, HF: heart failure, CAD: coronary artery disease, CKD: chronic kidney disease, DM: diabetes mellitus, aOR: adjusted odds ratio, HR: hazard ratio, 95% CI: 95% confidence interval.
