# Supplementary material for: Oral Anticoagulation Choice and Dosage in Very Elderly Patients with Atrial Fibrillation
Source: J Cardiovasc Dev Dis. 2025 Feb 26;12(3):86. doi: 10.3390/jcdd12030086 (PMC11943373; doi:10.3390/jcdd12030086)
Supplement: Supplementary file 1 [file jcdd-12-00086-s001.zip › jcdd-3459654-supplementary.pdf]

Supplementary Table S1. Type and dose of OAC prescribed at hospital discharge

Type of OAC (dose)	N (%)
Acenocoumarol	*164 (36.4)*
Full DOAC dose	*96 (21.3)*
Dabigatran 150 mg	10 (2.2)
Rivaroxaban 20 mg	41 (9.1)
Apixaban 5 mg	45 (10)
Reduced DOAC dose	*190 (42.3)*
Dabigatran 110 mg	49 (10.9)
Rivaroxaban 15 mg	62 (13.8)
Apixaban 2.5 mg	79 (17.6)
**Total**	**450 (100)**

Supplementary Table S2. Sensitivity analysis outcomes after adjustment for patients anticoagulation adherence during follow-up

	**Multivariate aHR* Cis (95%)**		**Multivariate aHR** Cis (95%)**
	Good OAC adherence	*DOACs at discharge*	*DOACs at discharge*
All-cause mortality	0.82 (0.58 -1.15)	0.86 (0.61–1.21)	0.79 (0.58-1.06)
CV mortality	0.82 (0.55-1.22)	0.79 (0.53–1.17)	0.76 (0.53-1.07)
Bleeding	0.52 (0.24-1.12)	1.30 (0.58-2.91)	1.50 (0.52-4.27)
Stroke	2.25 (0.30-19.30)	0.39 (0.27-1.05)	0.43 (0.17-1.08)
AF- or HF related hospitalization or CV death	0.88 (0.64-1.21)	0.90 (0.65-1.24)	0.84 (0.63-1.11)
	**Multivariate aHR* Cis (95%)**		**Multivariate aHR** Cis (95%)**
	Good OAC adherence	*Reduced DOACs dose at discharge*	*Reduced DOACs dose at discharge*
All-cause mortality	0.55 (0.35 -0.87)	1.05 (0.64–1.71)	0.96 (0.60–1.53)
CV mortality	0.45 (0.26 -0.75)	1.08 (0.59–1.98)	0.84 (0.47– 1.50)
Bleeding	1.99 (0.41-9.62)	1.42 (0.46-4.38)	1.32 (0.42– 4.16)
Stroke	1.74 (0.21-14.30)	1.38 (0.31-6.05)	1.24 (0.27– 5.70)
AF- or HF related hospitalization or CV death	0.55 (0.36-0.83)	0.86 (0.55-1.34)	0.79 (0.50– 1.22)

*Cox Regression analysis adjusted for Age, Gender, Smoking, BMI, Hx of Diabetes Mellitus, Hx of HF, Hx of CAD, Hx of Hypertension, Hx of CKD, Hx of Stroke, CHADS-VASc score, Proportion of Days Covered under OAC treatment >80% of total time of follow-up (i.e., good OAC adherence)

** Original Cox Regression analysis adjusted for Age, Gender, Smoking, BMI, Hx of Diabetes Mellitus, Hx of HF, Hx of CAD, Hx of Hypertension, Hx of CKD, Hx of Stroke, CHADS-VASc score
